# Regulation of MHC Class II-Peptide Complex Expression by Ubiquitination

**DOI:** 10.3389/fimmu.2013.00369

**Published:** 2013-11-13

**Authors:** Kyung-Jin Cho, Paul A. Roche

**Affiliations:** ^1^Experimental Immunology Branch, National Cancer Institute, National Institutes of Health, Bethesda, MD, USA

**Keywords:** MHC class II, March-I, ubiquitination, degradation, endocytosis, recycling

## Abstract

MHC class II (MHC-II) molecules are present on antigen presenting cells (APCs) and these molecules function by binding antigenic peptides and presenting these peptides to antigen-specific CD4^+^ T cells. APCs continuously generate and degrade MHC-II molecules, and ubiquitination of MHC-II has recently been shown to be a key regulator of MHC-II expression in dendritic cells (DCs). In this mini-review we will examine the mechanism by which the E3 ubiquitin ligase March-I regulates MHC-II expression on APCs and will discuss the functional consequences of altering MHC-II ubiquitination.

Major histocompatibility complex class II molecules (MHC-II) function by presenting processed antigens, derived primarily from exogenous sources, to CD4^+^ T-lymphocytes. MHC-II molecules thereby are critical for the initiation of the antigen-specific immune response. MHC-II is constitutively expressed by immune cells including B cells, monocytes, macrophages, and dendritic cells (DCs) and even non-hematopoietic cells can express MHC-II under inflammatory conditions. While each of these MHC-II-bearing cell types function as “professional” antigen presenting cells (APCs), DCs have received much attention as APCs since it is these APCs that are able to stimulate naïve antigen-specific CD4^+^ T cells. Tissue-resident DCs have often been referred to as the “sentinels of the immune system,” and it is their job to continuously sample their microenvironment by internalizing extracellular fluid and generating peptide-MHC-II complexes (pMHC-II) that can potentially interact with antigen-specific T cells (1). While resting (immature) DCs do express considerable amounts of pMHC-II on their surface, stimulation of DCs by a variety of inflammatory stimuli results in increased expression of pMHC-II at the plasma membrane by at least two mechanisms: (1) by increasing antigen proteolysis/peptide binding to MHC-II (2, 3) and (2) by promoting pMHC-II movement from intracellular antigen processing compartments to the cell surface (4, 5). Activation of DCs transiently increases MHC-II synthesis and increases macropinocytosis. However, within hours of the activation signal CIITA synthesis (and thus MHC-II synthesis) is severely reduced and macropinocytosis is terminated (6, 7). Together, these processes poise the recently activated DC to generate large amounts of pMHC-II with antigens derived from pathogens at the site of infection, thereby enhancing their ability to stimulate antigen-specific CD4^+^ T cells.

At steady-state, the rate of generation of pMHC-II complexes in immature DCs is equal to the rate of pMHC-II degradation. It is regulation of pMHC-II degradation that is the topic of this mini-review. Recently, it has been shown that ubiquitination participates in pMHC-II degradation (8, 9). MHC-II is ubiquitinated on a single conserved lysine in the cytoplasmic domain of the MHC-II β-chain present in mouse I-A and I-E molecules as well as human HLA-DR molecules, heretofore referred to as K225. The membrane-associated RING-CH-domain containing E3 ubiquitin ligase March-I is the sole E3 ligase responsible for the ubiquitination MHC-II in B cells and is the primary E3 ligase responsible for ubiquitination of MHC-II in DCs (10). March-I expression is highly enriched in secondary lymphoid tissues such as spleen and lymph node (11) and appears to be especially prominent in APCs such as B cells (10), DCs (12–14), and monocytes (15).

Expression of March-I leads to the down-regulation of several surface molecules including MHC-II, CD86, and transferrin receptor (TfR) (11, 16). In March-I-deficient B cells, MHC-II expression is much higher than in control B cells, and this effect was mediated by ubiquitination of K225 in the I-A β-chain (10). Gain-of-function experiments in which March-I was overexpressed in MHC-II-expressing HeLa-CIITA cells or human monocyte-derived DCs (MoDC) resulted in profound down-regulation of surface HLA-DR level in these cells (14, 16). Our own loss-of-function experiments revealed that expression of MHC-II was significantly higher on immature DCs isolated from March-I KO mice than on DCs isolated from WT mice. Essentially identical results were obtained using DCs isolated from MHC-II K_255_R ubiquitination-mutant mice, demonstrating that ubiquitination of MHC-II K225 by March-I regulates MHC-II surface expression (14).

Whereas March-I is constitutively expressed in resting professional APCs, March-I can be induced or repressed by different stimuli both *in vitro* and *in vivo*. Infection of mouse macrophages with *Francisella tularensis* induces the ubiquitin-dependent degradation of MHC-II by promoting IL-10-dependent March-I expression (17). IL-10 up-regulate March-I expression and MHC-II ubiquitination not only in mouse macrophages but also in human monocytes and mouse B cells (18, 19). Curiously, although most MHC-II-expressing APCs constitutively express March-I, interferon-gamma-treatment of monocytes, which leads to MHC-II expression, does not result in March-I expression unless the cells are also treated with IL-10, highlighting the complexity of March-I expression in APCs. Curiously, the ability of IL-10 to downregulate MHC-II expression in DCs is due to induction of March-I (15, 20). Perhaps more important than the up-regulation of basal March-I expression, March-I mRNA expression is significantly reduced when resting APCs are stimulated with toll-like receptor (TLR) signals such as LPS, PGN, poly (I:C) (16, 21). The March-I protein has a half-life of less than 30 min, potentially regulated by auto-ubiquitination (12), therefore the termination of March-I mRNA expression leads to a rapid drop in March-I protein levels (16). The reduction of March-I protein expression upon DC activation has profound consequences for MHC-II ubiquitination, for upon DC activation MHC-II ubiquitination is dramatically reduced (8–11, 21). As will be discussed below, it is the activation-induced termination of March-I expression that primarily regulates MHC-II surface expression in DCs.

The available evidence shows that ubiquitination by March-I is an important regulator of MHC-II degradation. Simple overexpression of March-I dramatically reduces the survival of MHC-II molecules in HeLa-CIITA cells and in B cells (10, 14, 16). In addition, studies in mutant mice have shown that surface MHC-II expression is higher and the half-life of MHC-II is significantly prolonged in B cells isolated from March-I KO mice as compared to WT mice (10). A similar role for human March-I in regulation of HLA-DR expression MoDCs has also been described (16). We have shown that surface pMHC-II complexes on March-I KO DCs or K_255_R ubiquitination-mutant immature DCs are considerably more stable than those in WT DCs and kinetic analyses demonstrated that ubiquitination directly affects the rate of degradation of surface pMHC-II (14). Limiting lysosomal proteolysis delays March-I-induced MHC-II degradation in DCs (9), suggesting that ubiquitinated MHC-II is degraded in late endosomes/lysosomes in these cell types.

In immature DCs, a relatively large pool of MHC-II is present in intracellular antigen processing compartments. During TLR-mediated DC activation many of these MHC-II molecules traffic to and accumulate on the plasma membrane (3). Maturation of DCs not only inhibits fluid-phase macropinocytosis in DCs (22, 23), but also inhibits the kinetics of MHC-II endocytosis from the cell surface in human MoDCs (6). The findings that MHC-II is (1) ubiquitinated in immature DCs, (2) internalizes efficiently in immature DCs, and (3) accumulates intracellularly in immature DCs (but not mature DCs) has led to speculation that ubiquitination regulates MHC-II endocytosis in DCs. It has been shown that anti-MHC-II mAb accumulate intracellularly in WT immature DCs but not in K_255_R ubiquitination-mutant immature DCs (8, 9, 14), a finding that is consistent with the hypothesis that ubiquitination regulates MHC-II endocytosis.

However, the role of ubiquitination in enhancing the kinetics of MHC-II internalization remains controversial. De Gassart et al. have reported that MHC-II internalization was reduced by 50% in MoDCs in which March-I expression was reduced by transfected siRNA (16). By contrast, our own studies in both human and mouse DCs have shown that while MHC-II endocytosis is slightly more rapid in immature DCs than in mature DCs, there is no difference in the kinetics of MHC-II endocytosis in DCs from WT, March-I KO, and MHC-II K_255_R ubiquitination-mutant mice (14). Furthermore, analysis of March-I-deficient B cells revealed that the internalization rate of MHC-II in March-I KO B cells was similar to that in WT B cells, demonstrating that MHC-II ubiquitination is not required for internalization of MHC-II in B cells (10). We have also examined the kinetics of endocytosis of MHC-II in HeLa-CIITA cells expressing (or not) March-I. In agreement with our results in DCs, we found no difference in the rate of MHC-II endocytosis in HeLa-CIIA cells expressing GFP alone or GFP-March-I, demonstrating that ubiquitination of MHC-II does not affect the kinetics of MHC-II endocytosis in DCs (14). These data showing that ubiquitination does not affect MHC-II endocytosis rate are also consistent with similar types of experiments showing that ubiquitination profoundly affects the intracellular distribution of fibroblast growth factor receptor 1 and epidermal growth factor receptor but does so without affecting the kinetics of receptor endocytosis (24, 25).

Despite the significant effects of March-I on pMHC-II ubiquitination and pMHC-II localization, we do not yet have a clear understanding of how ubiquitination actually regulates the stability of pMHC-II complexes. Recently it has been found that the MHC-II polyubiquitin chain length is different in DCs and in B cells and that longer polyubiquitin chains (such as those present in DCs) promote more efficient MHC-II lysosomal targeting (26). How polyubiquitin chain length is regulated in APCs (whether by diminished ubiquitination or enhanced activity of deubiquitinating enzymes) remains to be determined. Clearly ubiquitination of MHC-II regulates MHC-II surface expression, and while our own data argues that ubiquitination does not directly affect pMHC-II endocytosis rate, the possibility exists that ubiquitination affects MHC-II surface expression by regulating the ability of pMHC-II to recycle back to the plasma membrane after endocytosis. pMHC-II complexes continuously internalize and recycle from early endosomes to the plasma membrane and back again (27). While analysis of internalization rate data has suggested that pMHC-II recycling rates are different in immature DCs and mature DCs (6), we have been unable to find direct experimental data to support this theory. We have recently shown that internalized pMHC-II enters into elongated Arf6^+^ Rab35^+^ tubular recycling endosomes and efficiently recycles back to the plasma membrane in HeLa-CIITA cells as well as in APCs (28). Although a direct link between pMHC-II recycling and ubiquitination has not been established, it is curious to note that overexpression of March-I promotes the re-distribution of MHC-II from early endocytic compartments to terminal lysosomes (16) and also that MHC-II co-localizes with recycling TfR^+^ endosomes more in March-I-deficient B cells as compared to WT B cells (10). Furthermore, overexpression of the related MARCH family member MARCH-8 alters the itinerary of proteins internalized by clathrin-independent endocytosis from recycling endosomes to terminal lysosomes (29), leading to the possibility that ubiquitination of pMHC-II by March-I serves to limit recycling and promote lysosomal degradation of pMHC-II complexes.

## Conflict of Interest Statement

The authors declare that the research was conducted in the absence of any commercial or financial relationships that could be construed as a potential conflict of interest.
